# Effects of Biofuel Crop Switchgrass (*Panicum virgatum*) Cultivation on Soil Carbon Sequestration and Greenhouse Gas Emissions: A Review

**DOI:** 10.3390/life12122105

**Published:** 2022-12-14

**Authors:** Jian Bai, Laicong Luo, Aixin Li, Xiaoqin Lai, Xi Zhang, Yadi Yu, Hao Wang, Nansheng Wu, Ling Zhang

**Affiliations:** 1 Jiangxi Provincial Key Laboratory of Silviculture, College of Forestry, Jiangxi Agricultural University, Nanchang 330045, China; 2 National Innovation Alliance of *Choerospondias axillaris*, Nanchang 330045, China

**Keywords:** biofuel crops, carbon sequestration, greenhouse gas emissions, net ecosystem CO_2_ exchange, phytoremediation

## Abstract

Under the macroenvironmental background of global warming, all countries are working to limit climate change. Internationally, biofuel plants are considered to have great potential in carbon neutralization. Several countries have begun using biofuel crops as energy sources to neutralize carbon emissions. Switchgrass (*Panicum virgatum*) is considered a resource-efficient low-input crop that produces bioenergy. In this paper, we reviewed the effects of switchgrass cultivation on carbon sequestration and greenhouse gas (GHG) emissions. Moreover, the future application and research of switchgrass are discussed and prospected. Switchgrass has huge aboveground and underground biomass, manifesting its huge carbon sequestration potential. The net change of soil surface 30 cm soil organic carbon in 15 years is predicted to be 6.49 Mg ha^−1^, significantly higher than that of other crops. In addition, its net ecosystem CO_2_ exchange is about −485 to −118 g C m^−2^ yr^−1^, which greatly affects the annual CO_2_ flux of the cultivation environment. Nitrogen (N) fertilizer is the main source of N_2_O emission in the switchgrass field. Nitrogen addition increases the yield of switchgrass and also increases the N_2_O flux of switchgrass soil. It is necessary to formulate the most appropriate N fertilizer application strategy. CH_4_ emissions are also an important indicator of carbon debt. The effects of switchgrass cultivation on CH_4_ emissions may be significant but are often ignored. Future studies on GHG emissions by switchgrass should also focus on CH_4_. In conclusion, as a biofuel crop, switchgrass can well balance the effects of climate change. It is necessary to conduct studies of switchgrass globally with the long-term dimension of climate change effects.

## 1. Introduction

In recent years, global climate change, especially global warming, has attracted widespread attention from all walks of life worldwide. Internationally, the United Nations Framework Convention on Climate Change (UNFCCC) reached The Paris Agreement at the Paris Climate Change Conference. The Paris Agreement aims to limit the increase in global average temperatures to 2 °C from pre-industrial periods and to limit temperature increases to 1.5 °C to constrain global temperature rise as soon as possible. The leading cause of global warming is the increase in greenhouse gases produced by human activities [1,2]; the main greenhouse gases are carbon dioxide (CO_2_), nitrous oxide (N_2_O), and methane (CH_4_) [3].

To limit temperature growth to 2 °C, the remaining global cumulative CO_2_ emissions should not exceed 400–1000 Gt by the end of the century. Therefore, how to effectively control carbon emissions, especially human-induced carbon emissions, has attracted more attention from the international community. For non-CO_2_ greenhouse gases, CH_4_ and N_2_O are of concern. According to the global warming potential (GWP) calculation, the GWP of CH_4_ is about 23–25 times that of CO_2_ and the GWP of N_2_O is about 296 times that of CO_2_ [4].

**Figure 1:**
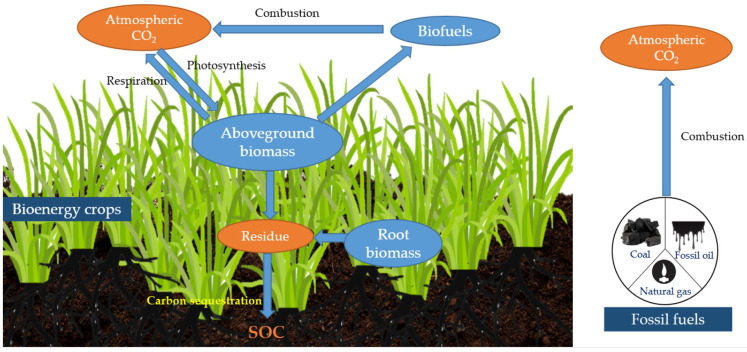
Carbon turnover process of biofuel crops vs. fossil fuels.

Carbon emitted from fossil fuels since the industrial revolution is about 420 Gt C [5]. Globally, CH_4_ and N_2_O emissions from agriculture exceed 610 million tons per year, accounting for 12% of total emissions [6]. Therefore, reducing agriculture’s carbon emissions is a crucial issue. Biofuel crops are mainly perennial (herbaceous or woody) that improve soil quality, promote nutrient cycling and carbon fixation, and can produce large quantities of high-carbon biomass. Compared with fossil fuels, biofuel crops have greater advantages in energy utilization [7] (Figure 1). Furthermore, biofuel crops require less maintenance and input and can be adapted to marginal soils. Eggelston et al. [8] showed that 300–1300 Mt C fossil fuels can be replaced if 10–15% of agricultural land is used to grow biofuel crops. Moreover, under the circumstances, CH_4_ emissions from agriculture can be reduced by 15–56% and N_2_O emissions can be reduced by 9–26%.

Switchgrass (*Panicum virgatum*), a species of grass in the family Poaceae, is an adaptable perennial herbaceous C4 plant native to North America. It is mainly distributed in several countries south of 55° north latitude. There are two ecotypes, including upland and lowland. In general, lowland types, which can grow up to more than 3 m, have larger biomass than upland types [9]. The tillers of the upland ecotype are usually shorter and better adapted to cold and dry habitats [10]. Since the mid-1980s, switchgrass has been mainly used as a renewable biofuel source for research. So far, switchgrass has been used in various forms of biofuel conversion processes, including cellulosic ethanol production, biogas, and direct combustion [11,12]. As a biofuel source, switchgrass has a lower demand for fertilizers and pesticides, which allows switchgrass to produce good yields on the land of the best part of soil types [13]. The climate benefits of biofuels are mainly manifested in (1) the use of alternative fossil fuels; (2) reducing greenhouse gas emissions during biofuel production, mainly through soil C accumulation and avoidance of greenhouse gas emissions. This paper discusses the potential contribution of switchgrass in carbon sequestration and greenhouse gas emission reduction. The future application and study of switchgrass are discussed and prospected.

## 2. Carbon Sequestration by Switchgrass

**Figure 2:**
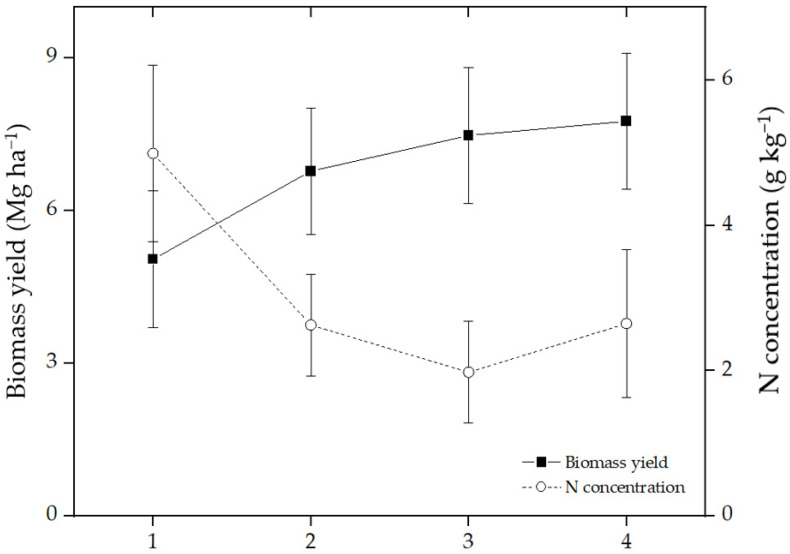
Average biomass yield and N concentrations in biomass of switchgrass across locations in the USA. Data were replotted from Hong et al. [20].

Soil and plant carbon sequestration is a practical way to mitigate CO_2_ emissions [14,15]. As early as the 1990s, Ma et al. [16] studied the effects of soil management measures, including nitrogen (N) application, row spacing, and harvest frequency, on carbon sequestration in switchgrass fields established for 2–3 years. The results found that the soil management measures of switchgrass did not change the soil carbon concentration. Interestingly, they compared the soils of the switchgrass and their adjacent fallow soils that had been established for some time (10 years). The results showed that the soil organic carbon (SOC) of the switchgrass was significantly higher than that of the fallow land; the SOC of the 0–15 cm soil increased by 44.8% and in the 15–30 cm soil it increased by 28.2% [16]. Therefore, switchgrass soil can store more soil carbon, although detecting it may take several years. Carbon sequestration in the switchgrass field does not occur only in the topsoil. Liebig et al. [17] show that switchgrass soils below 30 cm can also effectively sequester SOC. C stored in deep soils is not prone to mineralization and erosion. According to a four-year measurement, after four growing seasons, the SOC produced by switchgrass is 9.45 Mg ha^−1^ [18]. Different ages of switchgrass have different changes in the underground 30 cm SOC. A prediction from Anderson et al. [19] of net changes in SOC indicated that the change of switchgrass to the underground SOC increases with time and the switchgrass cultivated for 15 years increases by about 6.49 Mg ha^−1^ (Table 1). Hong et al. [20] found that the biomass of switchgrass fields across locations in the USA increased significantly in the first three years after the establishment (Figure 2). The total yield in the third and fourth years was similar (Figure 2). At a soil depth of 1 m, the SOC of switchgrass soil was 9.4% higher than that of farmland and 8.1% higher than that of *Andropogon gerardi,* while the quality of soil N is basically the same as that of farmland [21].

Although the effects of switchgrass soil management measures on soil carbon sequestration did not have a significant effect in the study of Ma et al. [16], some studies have shown that fertilizer management measures and harvesting methods have essential effects on switchgrass carbon sequestration [22,23,24,25]. On the Conservation Reserve Program (CRP) land dominated by switchgrass in South Dakota, there is no benefit if the N applied exceeds 56 kg ha^−1^ [24]. The application of NH_4_NO_3_ and manure can effectively increase switchgrass’s soil carbon sequestration, especially at soil depths of 30–90 cm [23]. Switchgrass is a perennial herb whose roots can grow deep in the soil. It has considerable root biomass, which is more than the aboveground biomass [16]. The root biomass of switchgrass in different soil types at different depths is shown as follows (Table 2). Zan et al. [26] showed that switchgrass has a biomass 4–5 times that of maize and can store 2.2 Mg C ha^−1^ yr^−1^. Liebig et al. [17] found that the cumulative rate of C was 1.1 Mg C ha^−1^ yr^−1^, most of which occurred at depths of 30 cm underground. Tulbure et al. [27] used RF (Random Forest packet in R) to analyze the effects of multiple factors such as fertilizer, genetics, and precipitation on yield. The results showed that the total variance of RF interpretation was 75%, with N fertilizer being the most important explanatory variable, followed by genetics, precipitation, and management measures.

## 3. Net Ecosystem CO_2_ Exchange of Switchgrass

Net ecosystem CO_2_ exchange (NEE) is the result of imbalances between total primary production (GPP) and ecosystem respiration (Re), which can affect carbon dynamics and budgets [29]. A better understanding of switchgrass’s NEE changes will help assess switchgrass’s potential for climate change mitigation. Some NEE of biofuel crops are shown below (Table 3). Zeri et al. [30] found that switchgrass has a stronger carbon sink capacity at the initial establishment stage than *Miscanthus* × *giganteus* (giant miscanthus, a sterile hybrid of *Miscanthus sinensis* and *Miscanthus sacchariflorus*). Compared with corn, switchgrass absorbs more carbon. The NEE of switchgrass is −336 ± 40 g C m^−2^ and that of corn is 64 ± 41 g C m^−2^ [31]. From 2012 to 2013, the analysis of the NEE of switchgrass [32,33,34] showed that it had a stronger carbon sink capability than sorghum land. This may be because that switchgrass has a net carbon sink of about 4–5 months (April/May–August) and sorghum has only 3 months of net carbon sink (June–August).

Surprisingly, in a study by Zenone et al. [37], the switchgrass field did not exist as a carbon sink but produced CO_2_ emissions. However, their measurements were only carried out for 2 years. In contrast, in the 4-year study [18], CO_2_ can be fixed each year and NEE stabilized at higher values from the second year, although the cumulative biomass in the first year was relatively low. Zenone et al. [37] and Virgilio et al. [18] conducted studies on a newly established switchgrass field. For mature switchgrass fields, Eichelmann et al. [35] conducted two years of data collection and found that NEE is 106 ± 45 g C m^−2^ in the first year, which was represented as a carbon source, while the NEE in the second year was −59 ± 45 g C m^−2^, which was manifested as a carbon sink. Previous four-year studies of mature switchgrass fields [36] showed that the first three years of switchgrass forests served as a sink of net CO_2_, while the following year became a source of CO_2_ emissions. These results suggest that switchgrass may be able to act as a powerful carbon sink in its establishment years, then its benefits will be reduced or even transformed into a carbon source.

## 4. CH_4_ Flux as Affected by Switchgrass Cultivation

The soil can be either a sink or a source of CH_4_. Some studies [38,39] have shown that forest and grassland soils are the primary consumers of CH_4_ thanks to methane oxidation bacteria in soils. However, some agronomic and fertilization measures reduce the function of CH_4_ oxidation in soils [40]. This is because these measures can change the N state of the soil, temperature, water content, and other factors [41].

There is less research on CH_4_ emissions from switchgrass cultivation but more on the biogas production of switchgrass. However, a recent study [28] shows that under the condition of planting switchgrass, CH_4_ consumption per year is 39–47% less than that of unplanted plots. As far as CH_4_ is concerned, planting switchgrass is detrimental to CH_4_ emission reductions. After all, the GWP of CH_4_ in 100 years is about 25 times that of CO_2_. The CH_4_ flux produced by planting switchgrass should be counted in the carbon budget. In future studies, more measurements of CH_4_ data will be needed to pay more attention to CH_4_ emissions.

## 5. N_2_O Emission from Switchgrass Soil

### 5.1. N_2_O Emission of Switchgrass Soil with N Addition

**Figure 3:**
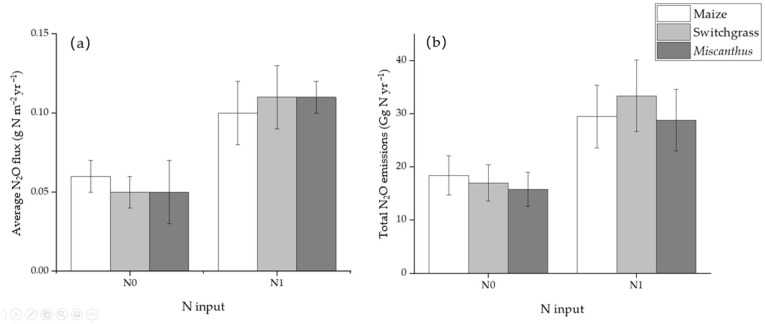
Estimated average N_2_O fluxes (**a**) and total N_2_O emissions (**b**) at different N input levels (N0: 0 g N ha^−1^ yr^−1^ N1: 67 g N ha^−1^ yr^−1^) in the conterminous United States. Data were replotted from Qin et al. [44].

Using winter legumes as nitrogen (N) sources is an N addition measure in agriculture. However, research shows that winter legumes will not increase the yield, cellulose, lignin, and hemicellulose concentration of switchgrass [42]. It may be that legumes are not conducive to use as the main nitrogen source of switchgrass. The primary source of N_2_O is the microbial processes of nitrification and denitrification in the soil and it is easy to increase N_2_O emissions through N input to the soil. Crutzen et al. [43] argue that N_2_O emissions caused by N-fertilizers required for the production of energy plants may offset the effects of energy plants in reducing the greenhouse effect or even exacerbate the greenhouse effect. Qin et al. [44] estimated the potential greenhouse gas emissions of the bioenergy ecosystem using the biogeochemical model AgTEM, a generic agroecosystem model with vegetation specific parameters characterizing specific crop structures and processes [45]. The results show that the N_2_O flux of switchgrass and *Miscanthus* in the United States is equivalent to that of corn (Figure 3). According to the crop type and nitrogen application rate, the N_2_O flux is about 0.05–0.11 g N m^−2^ per year [44].

However, Wile et al. [46] studied greenhouse gas emissions such as N_2_O from N applications to biofuel plants. The results showed that the annual cumulative N_2_O emissions of switchgrass cultivation systems were low and would not offset the benefits of using these biofuel feedstocks instead of fossil fuel energy. Wile et al. [46] argue that N fertilizers increase N_2_O emissions, but that increases in plant biomass can offset these increases. Similarly, Nikiema et al. [47] found that N fertilizer (0 to 112 kg N ha^−1^) had no effect on the N_2_O emission of switchgrass but increased its yield. This indicated that the N application reduced GHG emissions per unit plant biomass. Schmer et al. [48] determined the greenhouse gas fluxes of switchgrass during the growing season in the Great Plains north of Mantan and found that the application of N fertilizer affected the N_2_O flux during the growing season but did not affect the flux of CO_2_ and CH_4_. However, Ruan et al. [49] demonstrated that applying N fertilizer to mature switchgrass had little effect on yield but increased N_2_O emissions (Table 4). McGowan et al. [50] applied different levels of N treatment to switchgrass. The results showed that N fertilizer application higher than switchgrass demand could lead to large N_2_O emissions, negatively affecting GHG emissions. Therefore, how to apply N fertilizer reasonably is a crucial problem. It is necessary to ensure that the benefits of N application on climate change mitigation will not be reduced by the N_2_O it generates. A meta-analysis from Wullschleger et al. [51] of switchgrass at 39 sites in 19 states of the United States found that the optimal N application amount of switchgrass was about 100 kg N ha^−1^.

The utilization efficiency of single and mixed cultivation of switchgrass is different. Duran et al. [52] showed that, compared with the mixed planting of switchgrass and local perennial grasses, the single planting of switchgrass increased N_2_O emissions and the potential nitrate–nitrogen leaching capacity of fertilized switchgrass plots. This may be because different varieties of herbs have different or partially overlapped demands for N, which provides a reasonable combination for a more effective use of N. Although perennial biofuel plants such as switchgrass will produce N_2_O during production, in general, perennial biofuel crops emit less N_2_O than annual crops during their establishment. According to Oates et al. [53], perennial systems produce much lower N_2_O emissions per unit of ground than annual cropping systems.

### 5.2. Microbial Mechanism of N_2_O Emission from Switchgrass Field

Soil microbial activities related to N_2_O emission mainly include nitrification and denitrification [54]. Ammonia oxidizing archaea (AOA) and ammonia oxidizing bacteria (AOB) are closely related to the first step of nitrification. The transformation of dissolved N into gaseous N in denitrification is mainly related to *nirK*, *nirS*, and *nosZ* genes [55]. AOA are more abundant than AOB in agricultural soil [56]. Pannu et al. [57] confirmed that AOB abundance in switchgrass fertilized plots was positively correlated with N_2_O emissions. They found that applying N fertilizer increased the quantity and activity of AOB, which would lead to an increase in N_2_O emissions from the fertilized plots. Similarly, mycorrhizal fungi, AOA, and AOB increased with N input [58]. The *nirS* and *nosZ* genes are related indicators of denitrification. The expression of *nirS* and *nosZ* genes in the N application area was significantly higher than that in the non-nitrogen application area, indicating that fertilization in these systems may change the denitrification activity and may lead to related nitrogen loss, without yield return [59].

### 5.3. Environmental Factors Affecting Soil N_2_O Emissions with Switchgrass

Switchgrass is a perennial plant that needs to be managed, and switchgrass production can be affected by changes in temperature and precipitation space [60]. Similarly, its production can be affected by temporal changes in climate. Behrman et al. [61] estimated the productivity of current and future switchgrass in the central and eastern United States. They predicted that future climate change would significantly affect the spatial distribution and productivity of switchgrass.

Not only is nitrogen added directly, but many environmental factors will also affect N_2_O emissions from switchgrasssuch as temperature and precipitation. For example, under the condition of fertilization, a large amount of precipitation and a high-temperature climate can create a substrate-rich environment with limited oxygen for microorganisms, which is a good promotion of some anaerobic microbial processes such as denitrification.

Duncan et al. [62] used quantile regression to evaluate the correlation of four environmental factors—NH_4_^+^, NO_3_^−^, soil temperature, and water-filled pore space (WFPS)—to the upper limit of N_2_O emissions from switchgrass soil. The results showed that these four factors were significantly and positively correlated with the upper limit of N_2_O flux. However, the regression slope of non-fertilized plots was generally lower than that of fertilized plots. Soil moisture is one of the main factors driving N_2_O emissions from soils. A study has shown that N_2_O is emitted optimally in the WFPS range of 70–80% [63]. In addition, changes in soil oxygen concentration caused by soil temperature also make soil denitrification extremely sensitive to increasing temperature [64].

Intercropping also affected N_2_O emissions from switchgrass. Pannu et al. [57] showed that intercropping alfalfa (70:30, switchgrass: alfalfa) reduced dry matter yield but increased N_2_O flux.

## 6. Application of Switchgrass Cultivation in Degraded Land

Land degradation is an important topic in the 21st century due to its impact on agricultural productivity, the environment, and food security. If the degraded land can be used for biofuel crops, it will benefit agriculture development. Switchgrass is found to have good tolerance to drought and flooding, so it is suitable for marginal land. Slessarev et al. [65] conducted a 10-year (2008–2018) study on degraded land after sandstorms in the United States to assess the impact of switchgrass on the deep organic carbon storage of the three marginal soils. The carbon storage of topsoil (approximately 0–30 cm depth) and subsoil (approximately 30–100 cm depth) in switchgrass areas were significantly higher than those in reference (*p* < 0.01). Moreover, the switchgrass cultivation can increase the operational taxonomic unit (OTU) richness of marginal land [28]. They found that when the marginal land was converted into switchgrass land, the Shannon index increased significantly over time and the community composition changed [28]. This result may indicate that switchgrass has caused the improvement in soil quality.

Long-term soil toxic trace metal pollution will change the soil organic matter and microbial community, thus destroying the ecosystem [66]. Phytoremediation refers to affecting pollutants through plant extraction, which concentrates pollutants (such as toxic trace metals) in the environment into plant tissues [67]. Phytoremediation has been used to repair degraded soil. For example, tomatoes (*Solanum lycopersicum*) are used to repair cadmium (Cd) contaminated soil. Caesar-137 and strontium-90 were removed from power using sunflowers (*Helianthus annuus*) after the Chernobyl accident [68]. Switchgrass is a metal accumulator used in agriculture as a phytoremediation strategy as well. This strategy has the advantage of disposing of contaminated sites without excavation. Switchgrass in situ promotes environmental pollutants’ decomposition, fixation, and removal. Switchgrass can accelerate the degradation of atrazine and other herbicides, can absorb toxic trace metals in soil, and has good agronomic characteristics and high biomass [69,70]. A large amount of biomass can be harvested through the annual harvest in several seasons. Therefore, it is feasible to use switchgrass for in situ extraction of toxic trace metals. Finally, the amount of toxic metals will be reduced so that the affected land can restore the natural ecosystem or be used for crops productively. Balsamo et al. [69] studied the enrichment of lead (Pb) by switchgrass and timothy grass (*Pheum pretense*) and found that when the soil Pb concentration was 120 mg kg^−1^, the Pb content in switchgrass leaves was 0.028 ± 10% of the dry weight of leaves. In other words, assuming the soil Pb concentration is the same, based on the 7.5 t ha^−1^ harvest yield (Table 3), about 0.02 t lead can be removed by switchgrass every year, which is a considerable number. A study showed that switchgrass has medium tolerance to Cd and that a low concentration of Cd (100–175 μM) promoted the growth of switchgrass [71]. Fertilization can be used to improve the absorption capacity of switchgrass to toxic trace metals. The study showed that plants receiving high nitrogen had significantly the largest leaf dry mass and the highest Pb concentration [72]. Chelating agents can also promote the absorption of Pb by switchgrass. When NTA (nitrogenous acid) and APG (aluminum polyglucoside) were applied together, the Pb concentration in switchgrass leaves was more than doubled [73].

Moreover, the contaminated biomass harvested can be used as raw material for biofuel production. Cellulose, hemicellulose, and pectin can be decomposed into glucose or other sugars by enzymes and then bio-ethanol can be produced by yeast fermentation [74].

## 7. Conclusions and Future Prospects

Overall, most studies believe that changes in SOC or NEE caused by switchgrass cultivation have a positive effect on climate change. Although short-term research shows that SOC can be significantly increased by perennial biomass production, a long-term measurement is required to assess the dynamics of SOC.

N_2_O emissions from switchgrass are lower than from most other perennial grasses and annual crops. For the GHGs (mainly N_2_O) directly emitted during switchgrass cultivation, more effective fertilizer utilization strategies must be developed and used. N_2_O emissions can be reduced by estimating crop N demand and by improving N-use efficiency through timely fertilization.

Growing energy demands and concerns about climate change drive the use of energy plants, but, even so, the biofuel plant land cannot be developed unbridled. It is not suitable to develop biofuel plants with land that could be planted with large amounts of food, thus posing a danger to food security. Furthermore, more consideration should be given to using some marginal land to develop and grow biofuel plants such as switchgrass. It can be considered to establish switchgrass on some lands with highly toxic trace metals to simultaneously achieve the goal of carbon sequestration and soil restoration.

To develop an optimal cultivation strategy, future studies need to pay more attention to the relationship between fertilization, yield, and C and N loss. It is appropriate to consider the efficient breeding of switchgrass in order to establish switchgrass fields in a shorter time. Meanwhile, CH_4_ is a non-negligible carbon debt, which should be taken into account when calculating carbon loss data. Moreover, a comprehensive GHG budget and explicit spatial modeling of soil and plant carbon stocks should be considered to fully assess the impact of the large-scale transformation of these prairie sites.

## Figures and Tables

**Table 1:** Projected net changes in SOC (Mg C ha^−1^) in the top 30 cm of soil under biofuel crops of various ages. Adapted from Anderson et al. [19].

	Net Change in SOC (Mg ha^−1^ per 30 cm)		
Ages (Year)	Switchgrass	Sugarcane	*Miscanthus*
5	2.66	−34.21	2.31
10	4.64	−31.57	2.97
15	6.49	−28.93	3.63

**Table 2:** The root biomass (kg m^−2^) of switchgrass in different soil types [28].

Depth (cm)	Clay Loam	Sandy Loam
	Root Biomass (kg m^−2^)	
0–20	7.28 ± 0.44	7.44 ± 0.39
20–40	2.66 ± 0.10	1.97 ± 0.43
40–60	1.75 ± 0.07	1.84 ± 0.33
60–80	1.25 ± 0.08	3.23 ± 0.31
80–100	1.16 ± 0.07	2.26 ± 0.25

**Table 3:** Four energy crops’ net ecosystem CO_2_ exchange (NEE) of biofuel crops since 2005.

Location	Year	Crop	NEE (g C m^−2^ yr^−1^)	Citation
Urbana, IL, USA	2009	Switchgrass	−453 ± 20	[30]
		*Miscanthus*	−281 ± 30	
		Corn	−307 ± 40	
	2010	Switchgrass	−485 ± 20	
Guelph, ON, Canada	2014	Switchgrass	−336 ± 40	[31]
		Corn	64 ± 41	
Chickasha, OK, USA	2012	Switchgrass	−490 ± 59	[33]
		Sorghum	−261 ± 48	
	2013	Switchgrass	−406 ± 24	
		Sorghum	−330 ± 45	
Cadriano, Italy	2014–2016	Switchgrass	−733	[18]
Guelph, ON, Canada	2012	Switchgrass	−380 ± 25	[35]
	2013		−430 ± 30	
Ligonier, PA, USA	2005–2006	Switchgrass	−118	[36]
	2006–2007		−248	
	2007–2008		−189	

**Table 4:** Biomass yield of switchgrass and N_2_O emissions with different N addition treatments.

Location	Year	N Source	N Treatment (kg N ha^−1^ yr^−1^)	Yield (t ha^−1^ yr^−1^)	N_2_O Emissions (g N ha^−1^ yr^−1^)	Citation
Truro, NS, Canada	2009	NH_4_NO_3_	0	7.1	463	[46]
			40	6.6	345	
			120	7	933	
Mandan, ND, USA	2010	Urea	0	3.67	58.94	[48]
			67	4.47	184.29	
MI, USA	2009–2011	Urea	0	5.95	374.32	[49]
			28	6.91	512.34	
			56	7.85	698.45	
			84	7.62	964.03	
			112	7.72	1321.62	
			140	8.26	1806.78	
			168	7.82	2486.41	
			196	8.03	2867	

## Data Availability

Not applicable.
